# cAMP inhibits mammalian target of rapamycin complex-1 and -2 (mTORC1 and 2) by promoting complex dissociation and inhibiting mTOR kinase activity

**DOI:** 10.1016/j.cellsig.2011.06.025

**Published:** 2011-12

**Authors:** Jianling Xie, Godwin A. Ponuwei, Claire E. Moore, Gary B. Willars, Andrew R. Tee, Terence P. Herbert

**Affiliations:** a Department of Cell Physiology and Pharmacology, University of Leicester, The Henry Wellcome Building, University Road, Leicester LE1 9HN, UK; b Institute of Medical Genetics, School of Medicine, Cardiff University, Institute of Medical Genetics Building, Heath Park, Cardiff CF14 4XN, UK

**Keywords:** cAMP, mTORC1, mTORC2, TSC2, PKA

## Abstract

cAMP and mTOR signalling pathways control a number of critical cellular processes including metabolism, protein synthesis, proliferation and cell survival and therefore understanding the signalling events which integrate these two signalling pathways is of particular interest. In this study, we show that the pharmacological elevation of [cAMP]_i_ in mouse embryonic fibroblasts (MEFs) and human embryonic kidney 293 (HEK293) cells inhibits mTORC1 activation via a PKA-dependent mechanism. Although the inhibitory effect of cAMP on mTOR could be mediated by impinging on signalling cascades (i.e. PKB, MAPK and AMPK) that inhibit TSC1/2, an upstream negative regulator of mTORC1, we show that cAMP inhibits mTORC1 in TSC2 knockout (TSC2^−/−^) MEFs. We also show that cAMP inhibits insulin and amino acid-stimulated mTORC1 activation independently of Rheb, Rag GTPases, TSC2, PKB, MAPK and AMPK, indicating that cAMP may act independently of known regulatory inputs into mTOR. Moreover, we show that the prolonged elevation in [cAMP]_i_ can also inhibit mTORC2. We provide evidence that this cAMP-dependent inhibition of mTORC1/2 is caused by the dissociation of mTORC1 and 2 and a reduction in mTOR catalytic activity, as determined by its auto-phosphorylation on Ser2481. Taken together, these results provide an important insight into how cAMP signals to mTOR and down-regulates its activity, which may lead to the identification of novel drug targets to inhibit mTOR that could be used for the treatment and prevention of human diseases such as cancer.

## Highlights

► cAMP inhibits mTORC1 and mTORC2. ► cAMP causes the dissociation of mTORC1 and 2. ► cAMP inhibits mTORC1 independently of TSC2, Rheb, Rag GTPases, PKB, MAPK and AMPK. ► cAMP inhibits TORC1 via a PKA-dependent mechanism.

## 1 Introduction

Mammalian target of rapamycin (mTOR, also known as FRAP, RAFT or RAPT) is a Ser/Thr protein kinase that exists in two biochemically and functionally distinct multi-component complexes known as mTORC1 and mTORC2 [1,2]. mTORC1 couples nutrient availability with hormonal and growth factor signals to regulate metabolism, cell growth and proliferation [3,4], and phosphorylates a number of proteins involved in protein translation including ribosomal protein S6 kinases 1 and 2 (S6K1/2) and eukaryotic initiation factor 4E binding proteins (4EBPs) [5]. mTORC1 activity is regulated by numerous signalling pathways, many of which converge on tuberous sclerosis complex 1 and 2 (TSC1/2) [3]. TSC1/2 serves as a GTPases activating protein (GAP) for the small G-protein Rheb which in its GTP-bound state binds to and activates mTORC1. The association of Rheb and mTOR is promoted by amino acids availability [6]. It was reported that amino acids recruit mTORC1 to Rab7 positive vesicular structures containing Rheb via Rag GTPases and the trimeric “ragulator” [7,8]. mTORC2 is also important in the regulation of cell growth and proliferation and has been implicated in the organization of actin cytoskeleton [1]. These effects are mediated by mTORC2 dependent phosphorylation of AGC (for protein kinase A, G and C) kinase family members, such as protein kinase B (PKB), protein kinase C (PKC) and serum/glucocorticoid-induced kinase 1 (SGK1), which leads to their stabilization and full activation.

The second messenger cAMP (cyclic adenosine monophosphate) is yielded in response to a broad range of extracellular stimuli that act upon G-protein coupled receptors (GPCR) [9]. cAMP is synthesized by the action of adenylate cyclase (AC) and its degradation is mediated by the action of cAMP phosphodiesterases (PDEs) [10,11]. Most of the effects of cAMP are dependent upon the activation of its downstream effectors protein kinase A (PKA) [12] and exchange protein directly activated by cAMP (EPAC) [13]. Like mTOR, a diverse array of biological processes is regulated by cAMP [14]. Interestingly, there is good evidence of crosstalk between these two pathways. For instance, cAMP can either stimulate [15–20] or inhibit [21–26] mTORC1 depending upon cell type. This is likely through the activation or suppression of signalling transduction cascades upstream of mTORC1 such as the PKB [16,26–29], the MAPK (mitogen-activated protein kinase) [30] and the AMPK (AMP-activated protein kinase) pathways [23,25,31,32]. For example, glucagon, a GsPCR agonist that increase [cAMP]_i_, inhibits mTORC1 in rat hepatocytes [23,25]. This is coincident with an increase in AMPK phosphorylation on Thr172 [23,25], a positive regulator of TSC1/2 [33]. Of particular interest is that cAMP has anti-proliferative effects on cancer cells, which can be mediated by either targeting cell cycle regulatory molecules [24,34,35], or cellular signalling pathways pivotal to cell cycle progression [30]. It is possible that the anti-proliferative effects of cAMP are mediated, at least in part, by the inhibition of mTORC1 [24], as the over-activation of mTORC1 has been implicated in the development and maintenance of tumours [4].

In this report we demonstrate, in mouse embryonic fibroblasts (MEFs) and human embryonic kidney cells (HEK293), that cAMP signals to and inhibits mTORC1/2 via their dissociation and a decrease in mTOR intrinsic catalytic activity. We provide evidence that this is mediated by a novel PKA-dependent mechanism which is independent of TSC2, Rheb and Rag GTPases. This report provides new insights into how cAMP talks to mTOR and could lead to the discovery of new anti-cancer drug targets.

## 2 Materials and methods

### 2.1 Chemicals

All chemicals were purchased from Sigma-Aldrich (St. Louis, MO), unless specified.

### 2.2 Cell culture and treatments

TSC2^+/+^/p53^−/−^ MEFs (mouse embryonic fibroblasts), TSC2^−/−^/p53^−/−^ MEFs (kindly provided by Dr. David J. Kwiatkowski (Harvard Medical School, Boston, MA)) [36] and HEK293 (human embryonic kidney) cells were grown to approximately 80% confluence prior to treatment. MEFs were cultured in Dulbecco's modified Eagle's medium (DMEM) containing 25 mM glucose, supplemented with 10% (v/v) heat-inactivated foetal bovine serum (FBS) (Invitrogen, Carlsbad, CA), 100 μg/ml streptomycin, 100 units/ml penicillin sulphate, 100 units/ml neomycin, 50 μM β-mercaptoethanol and 1× non-essential amino acids. HEK293 cells were grown in DMEM containing 25 mM glucose, supplemented with 10% (v/v) heat-inactivated FBS, 100 μg/ml streptomycin, 100 units/ml penicillin sulphate and 100 units/ml neomycin. Cells were incubated at 37 °C in 5% CO_2_ and 95% air.

Prior to treatments (unless specified in the figure legends) the media were removed and the cells were washed twice and incubated with modified Krebs–Ringer bicarbonate buffer (KRB) (115 mM NaCl, 5 mM KCl, 10 mM NaHCO_3_, 2.5 mM MgCl_2_, 2.5 mM CaCl_2_, 20 mM HEPES, pH 7.4), supplemented with 0.5× MEM amino acids solution (50× stock), 0.5× MEM non-essential amino acids solution (100× stock) and 0.5×l-glutamine (100× stock).

After treatment (unless specified in the figure legends), cells were lysed by the addition of ice cold lysis buffer (1% (v/v) Triton, 10 mM β-glycerophosphate, 50 mM Tris–HCl, pH 7.5, 1 mM EDTA, 1 mM EGTA, 1 mM sodium orthovanadate, 1 mM benzamidine–HCl, 0.2 mM phenylmethylsulfonyl fluoride, 1 μg/ml each of leupeptin and pepstatin, 0.1% (v/v) β-mercaptoethanol and 50 mM NaF). Lysates were then centrifuged for 10 min at 16,000*g*. The supernatants were kept, and total protein concentrations were determined by Bradford assay (Bio-Rad, Hercules, CA). Normalized lysates were stored at − 20 °C until further analysis.

### 2.3 Adenoviral infection

Recombinant adenovirus expressing dominant negative AMPK was kindly provided by Dr. Ian Salt (University of Glasgow, UK) [37]. The cells were infected as previously described [38].

### 2.4 Transfection

HA tagged S6K1 (HA-S6K1) pRK7 vector was kindly provided by Prof. John Blenis (Harvard Medical School, Boston, MA, USA) [39], FLAG tagged Rheb (FLAG-Rheb) (plasmid 19996, Addgene, Cambridge, MA) and myc tagged mTOR (myc-mTOR) (plasmid 1861, Addgene, Cambridge, MA) were subcloned into the pRK7 vector. RagB^GTP^Q99L and RagC^GDP^S75L were described previously [40]. Cells were transfected by the CaCl_2_ method as previously described [41]. Cells were incubated for 24 h in DMEM prior to serum starvation for 18 h. Details of treatments are described in the figure legends.

### 2.5 Immunoprecipitation

The immunoprecipitation of mTORC1 and 2 complexes was essentially performed as previously described [40]. Briefly, after treatment, cells were lysed in 0.3% (w/v) CHAPS buffer (1 M HEPES pH7.5, 120 mM NaCl, 1 mM EDTA pH8, 10 mM sodium pyrophosphate, 10 mM β-glycerolphosphate, 50 mM NaF, 0.5 mM sodium orthovanadate, 0.3% CHAPS, 1 mM benzamidine-HCl, 0.2 mM phenylmethylsulfonyl fluoride, 1 μg/ml each of leupeptin and pepstatin). Protein lysates were then centrifuged for 10 min at 16,000*g*. The supernatants were kept, and total protein concentrations were determined by the Bradford assay. Lysates containing 1.2 mg of protein were incubated with anti-myc antibody (Sigma) for 16 h at 4 °C with rotation, followed by the incubation with protein-G sepharose beads for a further period of 2 h at 4 °C with rotation. Beads were washed three times with 0.3% (w/v) CHAPS buffer, and then resuspended in 2× Laemmli sample buffer.

### 2.6 SDS-PAGE and Western blotting

SDS-PAGE and Western blotting were performed as described previously [17]. Anti-PKCα was obtained from BD Transduction Laboratories (Oxford, UK). Anti-rpS6, GAPDH and Rheb were from Santa Cruz Biotechnology (Santa Cruz, CA, USA). Antiphospho-rpS6 Ser235/Ser236, rpS6 Ser240/Ser244, S6K1 Thr389, 4EBP1 Ser65, PKB Ser473, PRAS40 Thr246, ERK Thr202/Tyr204, AMPK Thr172, PKCα/βII Thr638/Thr641, mTOR Ser2481, mTOR Ser2448, as well as anti-S6K1, 4EBP1, PKB, AMPK, mTOR, RAPTOR, RICTOR, mLST8 and PRAS40 were purchased from Cell Signalling Technologies (Beverly, MA, USA).

### 2.7 Measurement of cAMP

Cells were grown in 24-well tissue culture plates. After treatment, buffer was removed and ice cold 0.5 M trichloroacetic acid was added. cAMP was extracted and determined using a radioreceptor assay with binding protein purified from calf adrenal glands as previously described [42]. [cAMP]_i_ was measured by interpolation of a standard curve and related to cellular protein, which was assessed by Bradford assay.

## 3 Results

### 3.1 cAMP inhibits insulin stimulated mTORC1 activation in HEK293 cells and MEFs

**Fig. 1:**
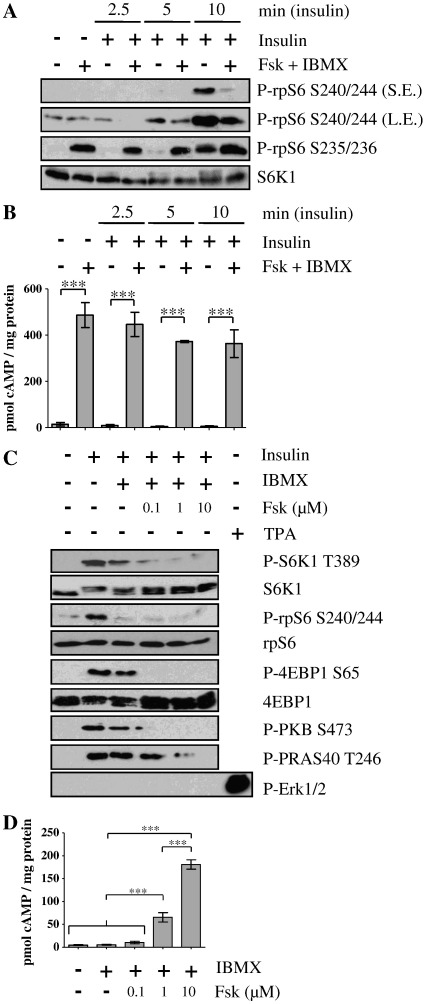
cAMP inhibits insulin signalling to mTOR. A) and B) HEK293 cells or C) and D) TSC2^+/+^ MEFs were serum starved in DMEM for 16 h before preincubated in KRB for 30 min, then incubated in KRB with or without forskolin (Fsk) (10 μM in A/B) plus IBMX (1 mM) for 30 min, before treatment with insulin (100 nM) or TPA (1 μM) for a further 30 min as indicated. A) and C) Proteins were resolved by SDS-PAGE and Western blotted using antisera against phosphorylated (P)- rpS6 Ser240/Ser244 (S240/244), Ser235/Ser236 (S235/236), P-S6K1 Thr389 (T389), P-4EBP1 Ser65 (S65), P-PKB Ser473 (S473), P-PRAS40 Thr246 (T246), P-Erk1/2, as well as S6K1, rpS6 and 4EBP1. S.E.: short exposure; L.E.: long exposure. [cAMP]_i_ levels in B) and D) were determined and expressed relative to cellular protein content, and shown as means ± SE; *n* = 3. In B), ***P < 0.001 versus time-matched control (no Fsk and IBMX) as indicated. There was no significant effect of insulin treatment on cAMP levels. For clarity, not all differences are shown. In C), ***P < 0.001 as indicated. Statistical analyses were by Bonferroni's test following one-way ANOVA. Immunoblots are representative of three independent experiments.

To determine the effect of cAMP on mTORC1 activation, HEK293 cells were pretreated with forskolin, a deterpene that activates adenylyl cyclase [43], and IBMX (3-isobutyl-1-methylxanthine) that suppresses PDEs [10]) to elevate [cAMP]_i_, (Fig. 1B) prior to the addition of insulin. As anticipated, forskolin and IBMX treatment caused the PKA-dependent phosphorylation of rpS6 (ribosomal protein S6) on Ser235/Ser236 [17], yet had no effect on the phosphorylation of Ser240/Ser244, a downstream target of S6K activation that is routinely used as a readout of mTORC1 activity [5] (Fig. 1A). Insulin alone led to a decrease in the electrophoretic mobility of S6K1, indicative of its phosphorylation and activation, and the rapid (within 5 to 10 min) phosphorylation of rpS6 on Ser240/Ser244. However, in cells pretreated with forskolin and IBMX (Fig. 1A), insulin-stimulated mTORC1 activation was suppressed as determined by the phosphorylation of rpS6 on Ser240/Ser244. These results demonstrate that, under these conditions, cAMP inhibits insulin-stimulated mTORC1 activation in HEK293 cells.

The effects of raising [cAMP]_i_ on insulin-stimulated mTORC1 activation was also ascertained in mouse embryonic fibroblasts (MEFs). In these cells, insulin also stimulated mTORC1 as determined by the phosphorylation of its downstream targets S6K1 on Thr389 and 4EBP1 on Ser65 and the phosphorylation of rpS6 on Ser240/Ser244 and a decrease in the electrophoretic mobility of S6K1 (Fig. 1C). In addition, insulin treatment led to an increase in the phosphorylation of PKB on Ser473, an indicator of its activation state [44]. PKB can activate mTORC1 through the phosphorylation and inactivation of TSC2, an upstream inhibitor of mTORC1 [45], and the phosphorylation of PRAS40 on Thr246 [46–48]. Indeed, insulin stimulated the phosphorylation of PRAS40 on Thr246 (Fig. 1C). The preincubation of cells with increasing concentrations of forskolin in the presence of IBMX, which was to dose dependently increase [cAMP]_i_ (Fig. 1D), suppressed insulin-stimulated mTORC1 activation as determined by the phosphorylation of S6K1 on Thr389, 4EBP1 on Ser65, rpS6 on Ser240/Ser244 and the electrophoretic mobility of S6K1. This paralleled a decrease in the phosphorylation of PKB at Ser473 and PRAS40 on Thr246 (Fig. 1C). Therefore, in MEFs, cAMP-dependent inhibition of mTORC1 may be mediated via the inhibition of PKB. The MAPK pathway is unlikely to be involved as insulin treatment of MEFs had no effect on the phosphorylation of ERK (extracellular signal-regulated kinase) (Fig. 1C).

### 3.2 cAMP inhibits mTORC1 independently of TSC2

**Fig. 2:**
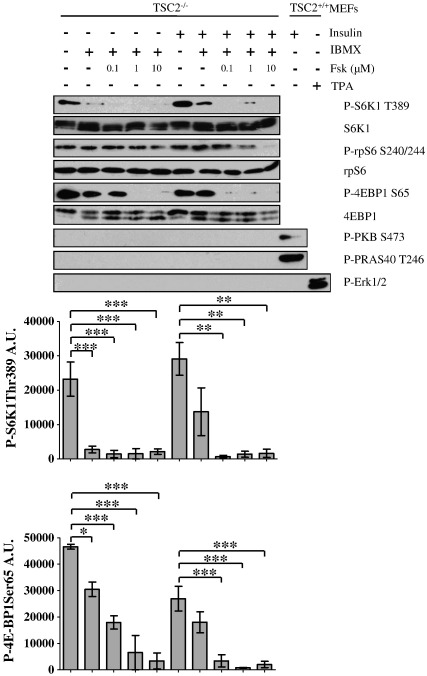
cAMP inhibits the activation of mTORC1 independently of TSC2. TSC2^−/−^ or TSC2^+/+^ MEFs were serum starved in DMEM for 16 h before preincubation in KRB for 30 min, followed by incubation in KRB with or without forskolin (0.1–10 μM) plus IBMX (1 mM) for 30 min. Cells were then treated with insulin (100 nM) or TPA (1 μM) for a further 30 min as indicated. Cell lysates were separated by SDS-PAGE and subjected to immunoblotting with antisera against phosphorylated (P)- rpS6 Ser240/Ser244 (S240/244), P-S6K1 Thr389 (T389), P-4EBP1 Ser65 (S65), P-PKB Ser473 (S473), P-PRAS40 Thr246 (T246), P-Erk1/2, as well as S6K1, rpS6 and 4EBP1. Levels of P-S6K1 Thr389 and P-4EBP1 Ser65 were quantified by densitometric analysis and are presented as arbitrary units (A.U.). Results shown are means ± SE; *n* = 3. *P < 0.05, **P < 0.01, ***P < 0.001 by Dunnett's test following one-way ANOVA. Immunoblots are representative of three independent experiments.

In order to explore the molecular mechanism by which cAMP inhibits mTORC1, we investigated whether cAMP could inhibit mTORC1 in cells deleted of TSC2. TSC2 knock-out (TSC2^−/−^) MEFs were pretreated with increasing concentrations of forskolin plus IBMX in the absence or presence of insulin (Fig. 2). mTORC1 is constitutively activated in these cells due to inactivation of the TSC1/2 complex [49]. Therefore, S6K1 and 4EBPs are constitutively hyper-phosphorylated in serum starved cells and insulin treatment is unable to further stimulate mTORC1. Importantly, incubation of these cells with forskolin/IBMX dose-dependently suppressed the phosphorylation of S6K1 (Thr389) and 4EBP1 (Ser65) (Fig. 2). This demonstrates that cAMP can inhibit mTORC1 independently of TSC1/2 and potentially reveals a novel and additional mechanism by which cAMP inhibits mTORC1. Moreover, no change in the phosphorylation state of PRAS40 was detected indicating that the suppression of mTORC1 activity is likely independent of PRAS40 in these cells (Fig. 2).

### 3.3 AMPK is not involved in the inhibition of mTORC1 by cAMP

**Fig. 3:**
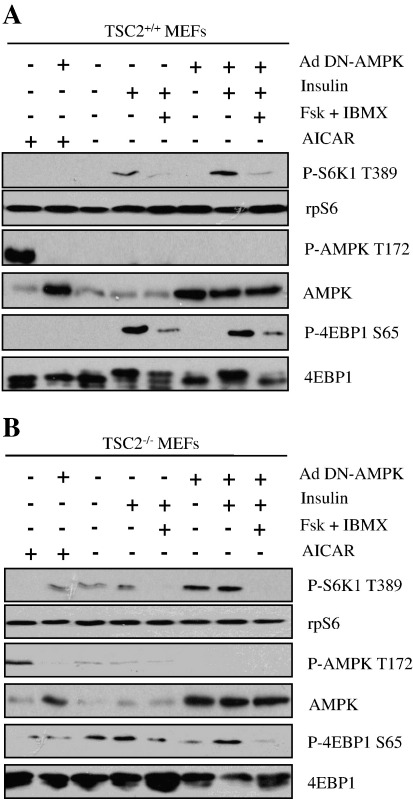
AMPK is not involved in the inhibition of mTORC1 by cAMP. A) TSC2^+/+^ and B) TSC2^−/−^ MEFs were infected with the recombinant adenovirus expressing dominant negative (DN) AMPK for 32 h. Cells were serum starved in DMEM for 16 h before preincubation in KRB for 30 min, and then treated with or without forskolin (10 μM) plus IBMX (1 mM) for 30 min, followed by a further 30 min incubation with insulin (100 nM) or AICAR (1 mM) as indicated. Cell lysates were separated by SDS-PAGE and subjected to immunoblotting with antisera against phosphorylated (P)- S6K1 Thr389 (T389), P-AMPK Thr172 (T172), P-4EBP1 Ser65 (S65), as well as AMPK, rpS6 and 4EBP1. All results are representative of three independent experiments.

Glucagon, a GsPCR agonist which augments [cAMP]_i_, can inhibit mTORC1 and this has been shown to correlate with the phosphorylation of AMPK [23,25]. Interestingly, AMPK can also block the activity of mTORC1 independently of TSC1/2 via the phosphorylation of RAPTOR [50]. To investigate whether the inhibitory effect of cAMP could be mediated by AMPK; TSC2^+/+^ (Fig. 3A) and TSC2^−/−^ (Fig. 3B) MEFs were infected with adenovirus expressing dominant-negative AMPK (AdDN-AMPK). The over-expression of DN-AMPK abolished AICAR (5-Aminoimidazole-4-carboxamide ribotide) induced phosphorylation of AMPK, yet cAMP was still able to suppress insulin-mediated mTORC1 activation in both TSC2^+/+^ and TSC2^−/−^ cells, as determined by the phosphorylation status of S6K1 and 4EBP1 (Fig. 3A and B). In addition, we were unable to detect any increase in AMPK phosphorylation in cells treated with forskolin and IBMX. Therefore, we conclude that an elevation in [cAMP]_i_ can inhibit mTORC1 independently of AMPK activity.

### 3.4 cAMP inhibits amino acid signalling to mTORC1

**Fig. 4:**
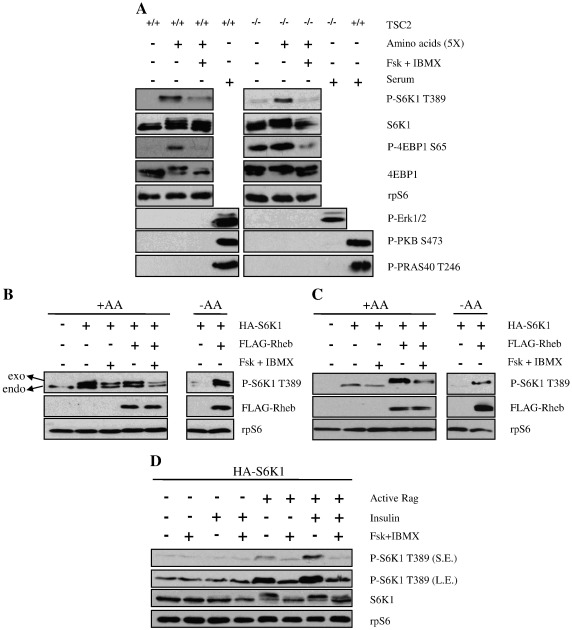
cAMP suppresses amino acids signalling to mTORC1. A) TSC2^+/+^ and TSC2^−/−^ MEFs were serum-starved in DMEM for 16 h before preincubation in KRB buffer supplemented with 20 mM glucose but in the absence of amino acids. Forskolin (10 μM) and IBMX (1 mM) were added 30 min post-preincubation, and then after another period of 30 min, cells were treated with amino acids (5× MEM) or serum (10%). Cells were lysed 30 min post-amino acids treatment. B) TSC2^−/−^ MEFs and C) HEK293 cells were co-transfected with FLAG-Rheb and HA-S6K1 for 48 h prior to the experiment. At 16 h before the experiment, cells were serum-starved in DMEM, and then preincubated in KRB buffer supplemented with 20 mM glucose and with (+ AA) or without (− AA) amino acids (5×) but with 20 mM glucose. At 30 min post-preincubation, cells were treated with forskolin (10 μM) and IBMX (1 mM) for 1 h before lysis. exo: exogenous S6K1, endo: endogenous S6K1. D) HEK293 cells were co-transfected with RagB^GTP^Q99L, RagC^GDP^S75L (constitutively active rag) and HA tagged S6K1 (HA-S6K1). At 32 h post-transfection, cells were serum-starved in DMEM for 16 h before preincubation in KRB supplemented with 20 mM glucose but in the absence of amino acids for 30 min, followed by the addition of forskolin (10 μM) plus IBMX (1 mM) for 30 min, and then further treated with insulin (100 nM) for another period of 30 min before lysis. Lysates were separated on SDS-PAGE and Western blotted using antisera against phosphorylated (P)- S6K1 Thr389 (T389), P-PKB Ser473 (S473), P-Erk1/2, as well as exogenous Rheb (FLAG-Rheb) rpS6 and S6K1. All results are representative of three independent experiments.

Amino acids alone are able to stimulate mTORC1 via a mechanism that bypasses TSC1/2 and Rheb [51]. To investigate whether cAMP can suppress mTORC1 activity driven by amino acids, TSC2^+/+^ and TSC2^−/−^ MEFs were incubated in the absence or presence of forskolin and IBMX prior to activation of mTORC1 by the addition of amino acids (Fig. 4A). As anticipated, the activity of mTORC1 was augmented in response to amino acids in both TSC2^+/+^ and TSC2^−/−^ cells as demonstrated by the phosphorylation of S6K1 (Thr389) and 4EBP1 (Ser65) and a decrease in the electrophoretic mobility of S6K1 and 4EBP1 (Fig. 4A). PKB, PRAS40 and ERK were not phosphorylated upon amino acids treatment. Interestingly, amino acid stimulated mTORC1 activation was attenuated in cells treated with forskolin/IBMX indicating that cAMP inhibits mTORC1 independently of TSC1/2 and Rheb (Fig. 4A).

The over-expression of Rheb can overcome the effect of amino acids depletion and constitutively activate mTORC1 [51,52]. Therefore, to provide additional evidence that the effect of cAMP on mTORC1 is independent on Rheb, TSC2^−/−^ MEFs (Fig. 4B) and HEK293 cells (Fig. 4C) were co-transfected with FLAG tagged Rheb (FLAG-Rheb) and HA tagged S6K1 (HA-S6K1). As anticipated, Rheb over-expression caused the constitutive activation of mTORC1, even in the absence of amino acids, as determined by the phosphorylation of S6K1 on Thr389 (Fig. 4B and C). Importantly, in Rheb over-expressing cells, the phosphorylation of S6K1 was attenuated in the presence of forskolin/IBMX in both TSC2^−/−^ MEFs and HEK293s (Fig. 4B and C). These results provide evidence that the inhibitory effect of cAMP on mTORC1 is independent of Rheb.

Rag GTPases are crucial for amino acids signalling to mTORC1 [7,53]. In order to study whether cAMP is exerting its inhibitory effect through Rag GTPases, active Rag GTPases (RagB^GTP^Q99L and RagC^GDP^S75L, as reported previously in ref. [7]) and HA-S6K1 were co-transfected in HEK293 cells, cells were incubated in KRB in the absence of amino acids. As amino acids play a permissive role in insulin signalling to mTORC1 [54], insulin alone was unable to induce the phosphorylation of S6K1 (Fig. 4D). In contrast, the over-expression of active Rag GTPases evoked the phosphorylation of S6K1, which was potentiated by the addition of insulin. Treatment of cells with forskolin/IBMX was still able to inhibit mTORC1 in the presence of active Rag GTPases (Fig. 4D). These results indicate that inhibition of mTORC1 by cAMP is independent of the role of active Rag GTPases in mTORC1 activation.

### 3.5 cAMP inhibits mTORC1 via PKA

**Fig. 5:**
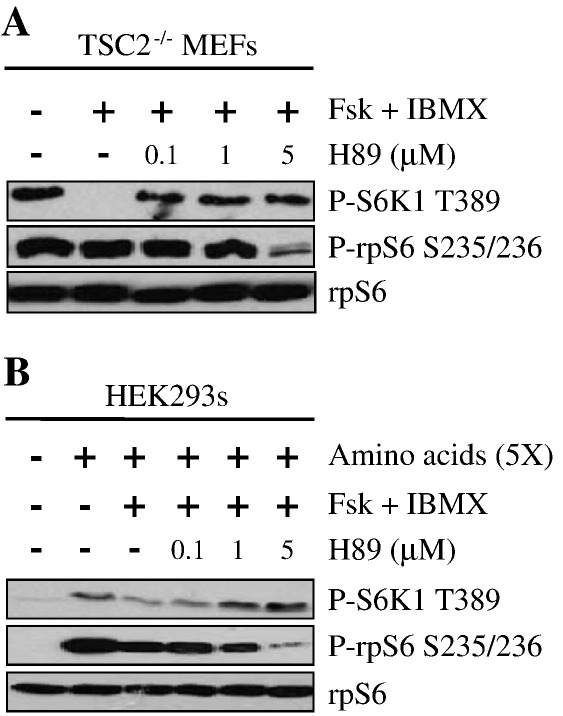
The inhibition of mTORC1 is PKA dependent. A) TSC2^−/−^ MEFs and B) HEK293 cells were serum-starved in DMEM for 16 h. For A), cells were incubated in KRB supplemented with 0.5× amino acids. For B), cells were incubated in KRB supplemented with 20 mM glucose but in the absence of amino acids. For both A) and B), after 30 min, cells were treated with increasing concentrations of H89, a PKA inhibitor, for 30 min, followed by treatment with forskolin (10 μM) and IBMX (1 mM) for 30 min, before stimulation with amino acids (5×) for a further period of 30 min (for B)). Proteins were resolved on SDS-PAGE and Western blotted using antibodies against phosphorylated (P)-S6K1 Thr389 (T389), P-rpS6 Ser235/236 (S235/236), and as a loading control, rpS6. All results are representative of three independent experiments.

PKA is a major effector of cAMP [55]. To determine whether the inhibitory effect of cAMP on mTORC1 is mediated through PKA, HEK293 cells were pretreated with the PKA selective inhibitor H89 prior to forskolin and IBMX treatment. As anticipated, amino acids stimulated the phosphorylation of S6K1 on Thr389 and this was inhibited by increased [cAMP]_i_ (Fig. 5). As expected, H89 dose-dependently inhibited the phosphorylation of rpS6 in Ser236/236 which is mediated by PKA [17]. Importantly this correlated with the recovery of S6K1 phosphorylation on Thr389 (Fig. 5), indicating that the inhibitory effect of cAMP on mTORC1 is likely mediated by PKA.

### 3.6 Increased [cAMP]_i_ causes dissociation of mTOR complexes and the inhibition of mTOR catalytic activity

**Fig. 6:**
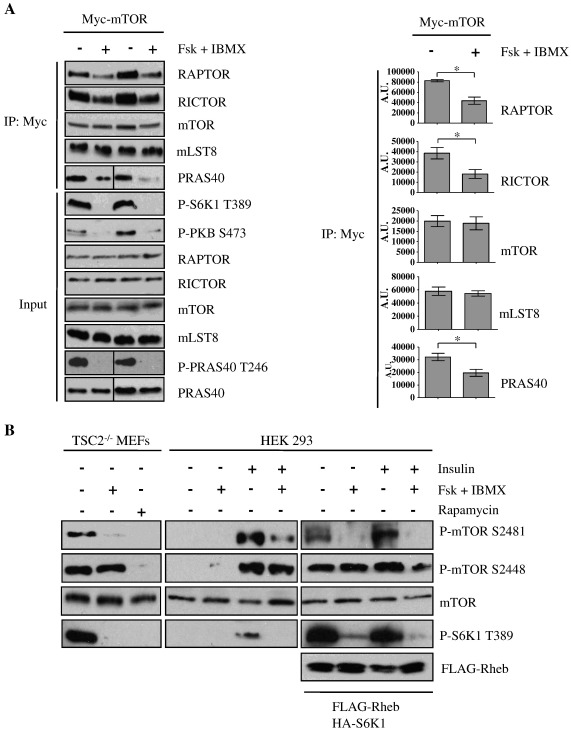
cAMP leads to dissociation of the mTOR complexes and inhibits the catalytic activity of mTOR. A) HEK293 cells were transfected with myc-tagged mTOR (myc-mTOR). At 32 h post-transfection, cells were serum-starved in DMEM for 16 h before preincubation in Dulbecco's-PBS for 30 min, and then stimulation with DMEM supplemented with 10% serum in the presence or absence of forskolin (10 μM) and IBMX (1 mM) for 1 h. Cell lysates were immunoprecipitated with anti-myc antibodies and immunoblotted as indicated. Levels of RAPTOR, RICTOR, mTOR, mLST8 and PRAS40 from immunoprecipitation were quantified by densitometric analysis. Results are means ± SE. *P < 0.05 by Student's paired *t*-test, *n* = 3 where the analysis was performed on the raw absorbance data. B) TSC2^−/−^ MEFs and HEK293 cells were serum-starved in DMEM for 16 h before preincubation in KRB for 30 min, then incubation in KRB with forskolin (10 μM) plus IBMX (1 mM) for 30 min, before further treatment with insulin (100 nM) for another period of 30 min. HEK293 cells were also co-transfected with FLAG-Rheb and HA-S6K 48 h prior to the experiment. Cell lysates were separated on SDS-PAGE and subjected to immunoblotting with antisera against phosphorylated (P)- mTOR Ser2481 (S2481), Ser2448 (S2448), S6K1 Thr389 (T389), and as loading controls, mTOR and exogenous Rheb (FLAG-Rheb). All results are representative of three independent experiments.

The results so far indicate that cAMP inhibits mTORC1 via the activation of PKA and that PKA inhibits mTORC1 independently of the classical regulatory inputs upstream of mTORC1. One possibility is that an elevation in [cAMP]_i_ causes the PKA-dependent dissociation of mTORC1. Therefore, we investigated whether the integrity of mTOR complexes is disturbed upon increases in [cAMP]_i_. For this purpose, HEK293 cells transfected with myc-tagged mTOR (myc-mTOR) were incubated with forskolin and IBMX prior to stimulation with serum, myc-mTOR and associated proteins were then immunoprecipitated from these cells. In serum repleted cells mTORC1 was active as determined by the phosphorylation status of S6K1. In addition, mLST8 and the mTORC1 specific components RAPTOR and PRAS40, and the mTORC2 specific component RICTOR were co-immunoprecipitated with myc-mTOR, indicating the integrity of mTORC1 and mTORC2 under these conditions. As expected, the preincubation of cells with forskolin/IBMX inhibited serum-stimulated mTORC1 activity, as determined by the phosphorylation status of S6K1. Moreover, preincubation of cells with forskolin/IBMX caused the dissociation of the mTORC1 specific component RAPTOR and PRAS40. In addition, forskolin/IBMX caused the dissociation of the mTORC2 specific component RICTOR from myc-mTOR. The association of mLST8 to myc-mTOR was unaffected (Fig. 6A). In conclusion, elevations in [cAMP]_i_ lead to the dissociation of mTORC1 and mTORC2.

To further investigate the molecular mechanism by which cAMP inhibits mTORC1, we monitored the auto-phosphorylation of mTOR on Ser2481, which has been shown to be a biomarker for intrinsic mTORC-specific catalytic activity [56]. In TSC2^−/−^ MEFs and insulin-stimulated or FLAG-Rheb transfected HEK293 cells, forskolin/IBMX treatment led to a reduction in the phosphorylation of mTOR on Ser2481 (Fig. 6B). Interestingly, the phosphorylation of mTOR on Ser2448, which has been reported to be mediated by S6K1 [57,58], was unaffected by cAMP, whereas rapamycin treatment led to a reduction in the phosphorylation of mTOR on both sites (Ser2481 and Ser2448) (Fig. 6B).

Taken together, these results suggest that cAMP inhibits mTORC1 via the perturbation of mTOR complex assembly and the reduction of mTOR catalytic activity.

### 3.7 cAMP inhibits mTORC2

**Fig. 7:**
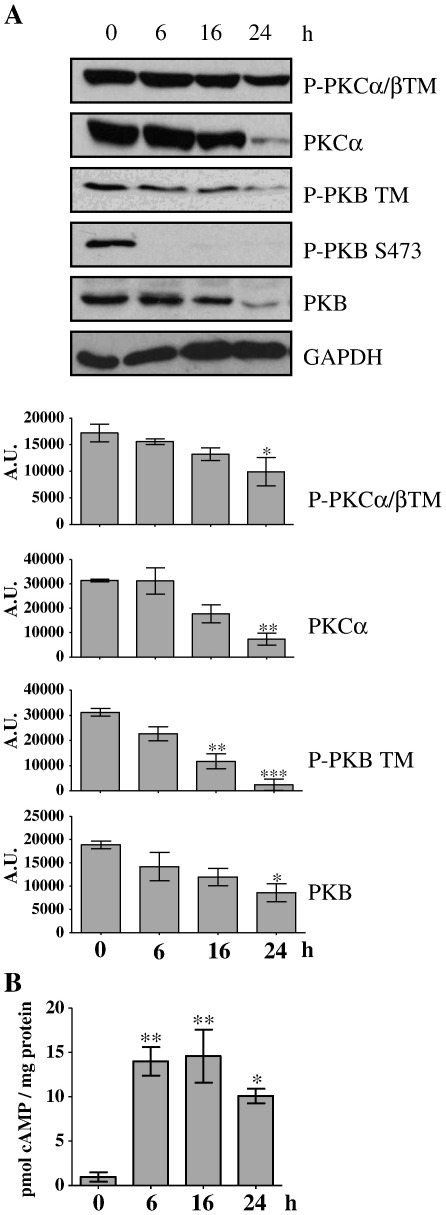
cAMP inhibits mTORC2. A) TSC2^+/+^ MEFs were maintained in growth media, treated with forskolin (10 μM) and IBMX (1 mM) for 6, 16 or 24 h. Cell lysates were resolved by SDS-PAGE, followed by Western blotting of phosphorylated (P)- PKCα/βII turn motif (TM), P-PKB TM, P-PKB Ser473 (S473), total PKCα and total PKB. GAPDH was used as a loading control. P-PKCα/βII TM, PKCα, P-PKB TM and PKB levels were quantified by densitometric analysis. B) [cAMP]_i_ levels were determined and expressed relative to cellular protein content. Results are means ± SE. *P < 0.05 **P < 0.01 ***P < 0.001 versus control (untreated) using one-way ANOVA followed by Dunnett's test, *n* = 3 where the analysis was performed on the raw absorbance data. Immunoblots are representative of three independent experiments.

As Forskolin/IBMX leads to the dissociation of mTORC2 and a decrease in mTOR activity it seems likely that elevations [cAMP]_i_ may also inhibit mTORC2. mTORC2 phosphorylates the turn motif on PKB, PKCα and PKCβII, resulting in their destabilisation and degradation [1]. mTORC2 is also responsible for the phosphorylation of PKB on Ser473 [59]. Therefore, to determine whether mTORC2 is also inhibited by [cAMP]_i_, TSC2^+/+^ MEFs were treated with forskolin and IBMX for up to 24 h and the phosphorylation and expression of PKB, PKCα and PKCβII were determined. Incubation of cells with Forskolin/IBMX caused a time-dependent reduction in turn motif phosphorylation on PKB, PKCα and PKCβII, and this paralleled a decrease in the protein levels of PKB and PKCα (Fig. 7A). These results provide evidence that elevation of [cAMP]_i_ not only inhibits mTORC1 (Figs. 1–4) but also inhibits mTORC2 (Fig. 7).

## 4 Discussion

cAMP and mTOR signalling pathways regulate fundamental cellular processes including metabolism, protein synthesis, proliferation and cell survival [60,61]. Therefore, it is of immense interest to understand the signalling events which integrate these two signalling pathways. In this study, we provide evidence that cAMP is able to inhibit the activation of mTORC1 and mTORC2 and this is likely mediated through the PKA-dependent disruption of the mTOR complexes 1 and 2 and the inhibition of mTOR catalytic activity.

Hormones and growth factors can activate mTORC1 via the PKB- and/or ERK/RSK-dependent phosphorylation of TSC2 and the inactivation of the TSC1/2 complex [3]. However, in some cell types, cAMP inhibits these signalling pathways [16,23,25–32] and hence inhibits hormone and growth factors activation of mTORC1. However, we demonstrate that cAMP can inhibit mTORC1 independently of these signalling pathways and independently of TSC1/2. Therefore, this work reveals an alternative and/or additional mechanism by which cAMP can inhibit mTORC1 in mammalian cells.

mTORC1 can also be regulated by the intracellular localization of Rheb independently of TSC1/2 [7,8]. For example, amino acids promote the co-localisation of mTOR with Rheb through a Rag GTPase dependent mechanism, which alone can activate mTORC1 [7,53]. Interestingly, the over-expression of Rheb can overcome the inhibitory effect of amino acids withdrawal and this is thought to be mediated by the inappropriate co-localisation of Rheb with mTORC1 [7,8]. It is unlikely that the inhibitory effect of cAMP on mTORC1 is caused by an alteration of the intracellular localization of Rheb as we show that forskolin/IBMX is able to reduce mTORC1 activity under conditions where Rheb or active Rag GTPases are over-expressed (Fig. 4). However, we cannot exclude the possibility that cAMP perturbs Rheb-GTP loading independently of TSC1/2, perhaps through impinging on its guanine nucleotide exchange factor.

Although the molecular mechanism by which cAMP inhibits mTOR is not fully understood, it is dependent on the activation of PKA (Fig. 5). One possibility is that PKA directly phosphorylates mTOR and inhibits mTOR kinase activity. Indeed, cAMP inhibits mTOR intrinsic catalytic activity as determined by the auto-phosphorylation state of mTOR on Ser2481 (Fig. 6B) and, interestingly, PKA has been reported to be associated with mTOR [62]. However, we and others have been unable to significantly phosphorylate mTOR *in vitro* with recombinant catalytic subunit of PKA (PKAc) (Xie, J. and Herbert, T.P., unpublished data, and [21]). On the other hand, it has been reported that glucagon, which elevates [cAMP]_i_, stimulates an increase in the phosphorylation of mTORC1 on Ser2448 in hepatocytes and this correlates with decreased mTORC1 activity [22]. However, this is unlikely to be the mechanism of inhibition as the mutation of this site has no effect on mTORC1 kinase activity [22,63]. Moreover, we could not detect any changes in the phosphorylation of mTOR on Ser2448 in response to elevated [cAMP]_i_ in either HEK293 cells or MEFs (Fig. 6B).

Importantly, we show that increased [cAMP]_i_ leads to the dissociation of both mTORC1 and 2 (Fig. 7A), which is known to inhibit both mTORC1 and mTORC2 activity [64–66]. For example, upon rapamycin treatment, mTORC1 dimerization is compromised and the complex is disassembled in a time-dependent manner [67]. However, whether complex dissociation as a result of increased [cAMP]_i_ follows the inactivation of mTOR or that mTOR inactivation follows the dissociation of the complex is unclear.

The phosphorylation of PRAS40 at Thr246 by PKB has been reported to promote mTORC1 activation through the dissociation of PRAS40 from mTOR [46–48]. To our surprise, the binding of PRAS40 to mTOR was reduced even though PRAS40 phosphorylation on Thr246 was ablated in response to cAMP (Fig. 7A). This raises doubt as to whether Thr246 can be used as an indicator of PRAS40 binding to mTORC1. As PRAS40 binds to RAPTOR within the complex [46,67,68], the dissociation of PRAS40 from mTOR upon forskolin/IBMX treatment is likely caused by the dissociation of RAPTOR.

During cancer development, the mTOR pathway is often abnormally up-regulated, which favours cancer cell survival, growth, replication, angiogenesis and metastasis [4]. Therefore, the inhibition of mTOR is a potential treatment for certain forms of cancer [69–71]. Similarly, cAMP negatively regulates cell cycle progression and cell motility in cancer cells, and therefore the augmentation of [cAMP]_i_ is a promising future cancer treatment [24,64,72–74]. It can be tempting to speculate that at least part of the anti-proliferative effect of cAMP is mediated through the inhibition of mTOR. However, cAMP can also target a number of cell cycle regulators such as p21^Cip1^, p27^Kip1^, Rb (retinoblastoma protein) [34,35] and CDK4 (cyclin D dependent kinase 4) [24]. Therefore, it is difficult to differentiate mTOR dependent and independent effects of cAMP on the control of proliferation.

In conclusion, we show that elevation of [cAMP]_i_ suppresses mTORC1/2 by promoting mTOR complex disassembly and inhibiting mTOR's intrinsic catalytic activity. These observations provide new insights into the crosstalk between cAMP and mTOR, which may also contribute to the design of novel mTOR inhibitors for future strategies in the fight against cancer.
